# The Link between Iron Turnover and Pharmacotherapy in Transplant Patients

**DOI:** 10.3390/nu15061453

**Published:** 2023-03-17

**Authors:** Marcin Delijewski, Aleksandra Bartoń, Beata Maksym, Natalia Pawlas

**Affiliations:** 1 Department of Pharmacology, Faculty of Medical Sciences in Zabrze, Medical University of Silesia, Jordana 38, 41-808 Zabrze, Poland; 2 Independent Researcher, 40-055 Katowice, Poland

**Keywords:** iron metabolism, graft, pharmacotherapy, anemia, iron deficiency, iron overload

## Abstract

Iron is a transition metal that plays a crucial role in several physiological processes. It can also exhibit toxic effects on cells, due to its role in the formation of free radicals. Iron deficiency and anemia, as well as iron overload, are the result of impaired iron metabolism, in which a number of proteins, such as hepcidin, hemojuvelin and transferrin, take part. Iron deficiency is common in individuals with renal and cardiac transplants, while iron overload is more common in patients with hepatic transplantation. The current knowledge about iron metabolism in lung graft recipients and donors is limited. The problem is even more complex when we consider the fact that iron metabolism may be also driven by certain drugs used by graft recipients and donors. In this work, we overview the available literature reports on iron turnover in the human body, with particular emphasis on transplant patients, and we also attempt to assess the drugs’ impact on iron metabolism, which may be useful in perioperative treatment in transplantology.

## 1. Introduction

Iron Fe^2+^/Fe^3+^ is the most abundant transition metal in the human body, and it plays an important role in the growth of the organism, energy metabolism and several physiological processes [1]. The iron content of the body normally reaches about 3 to 4 g, and exists mainly in the form of hemoglobin, iron-containing proteins (catalase, cytochromes, myoglobin) and transferrin-bound iron and is stored in the form of hemosiderin and ferritin. Iron is absorbed in the intestine, circulates in blood and cells and is stored mostly in the bone marrow, liver or spleen. The element is lost in shed skin cells, sweat and in the intestine. Menstrual iron loss leads to lower iron stores in women in comparison to men, and therefore women are more prone to become iron deficient [2,3,4]. Iron can also exhibit toxic effects on cells by catalyzing the reaction of free radical formation [1]. Moreover, iron plays a role in immune modulation, the mechanism of ischemia-reperfusion injury, and in the regulation of organ and graft functions [5].

Iron overload or anemia and iron deficiency are the result of impaired iron metabolism, in which a number of proteins, such as hepcidin and hemojuvelin, take part [1].

Iron deficiency occurs more commonly in individuals with renal, lung and cardiac transplants, while iron overload is more frequent in patients with hepatic transplantation [1,6]. Iron overload is related to poor prognosis in end-stage kidney and liver failure [5]. On the other hand, anemia and iron deficiency are connected with poor prognosis in patients with end-stage heart failure [5]. Moreover, iron deficiency prior to liver transplantation may be even a prognostic factor for the length of intensive care unit stay after operation [7].

Renal disease in the end-stage has also been stated to be associated with hyperferritinemia and the hepatic accumulation of iron [8], and before the introduction of erythropoiesis-stimulating agents in the recipients of renal graft, the iron overload frequency was about 28%. Either phebotomy or iron chelatation may be considered in these cases [5].

End-stage heart, liver and kidney diseases, and also chronic inflammatory conditions after organ transplantations, are often complicated by the anemia of chronic disease, when high hepcidin levels and proinflammatory cytokines lower iron delivery to the bone marrow cells and inhibit red blood cell production [5]. These effects may be associated with the interaction of hepcidin with ferroportin, taking part in the move of iron from the enterocytes to the blood. Hepcidin causes the internalization and degradation of ferroportin, which can lead, among other things, to the effect of “trapped iron in macrophages”.

When we consider the liver as a central store for iron in the body and the place where hepcidin is secreted as a result of increased plasma transferrin saturation and proinflammatory signals, the impact of liver diseases on iron turnover will be more significant. It is known that low transferrin as well as high ferritin levels are related to a worse outcome in patients with acute forms of liver failure [9]. In turn, chronic liver disease with reduced hepatocyte mass leads to the attenuated production of hepcidin and the augmented storage of iron in hepatocytes [5]. Nevertheless, in hereditary hemochromatosis, liver transplantation normalizes the secretion of hepcidin, preventing the regression of iron overload [10].

Perioperative challenges in transplantology, which are critically driven by iron, include such procedures as the selection of patients for transplantation, the management of immunosuppression, and the preservation of the function of graft prior to and after transplantation. Moreover, Schaefer et al. [5] suggest an active management of the status of iron in patients in the periprocedural period.

**Figure 1:**
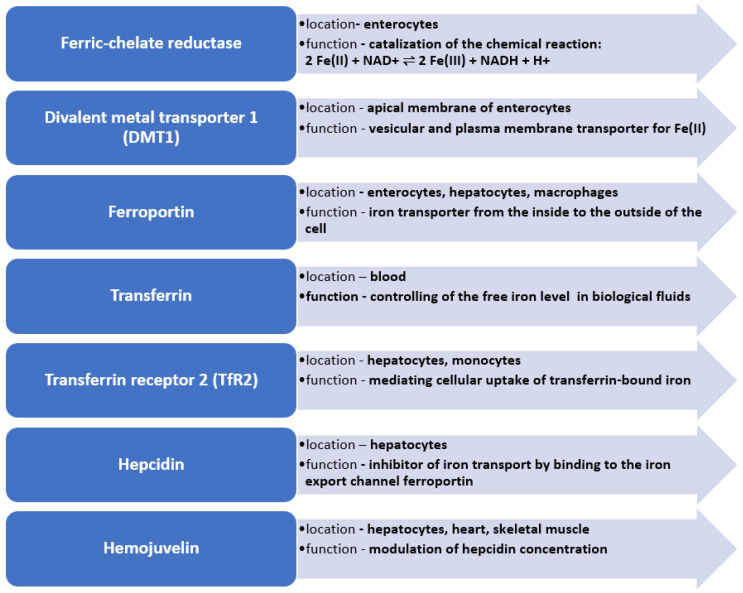
Key enzymes in iron metabolism in human body.

Disturbed iron metabolism in transplant recipients is often multifactorial. Causes should be found in the blood loss after transplantation, inflammatory processes, viral and microbial infections and drugs taken by those patients, especially immunosuppressive therapy (Table 1). Incorrect iron metabolism may cause a higher risk of anemia complications (Figure 1). Anemia may also increase the risk of cardiovascular events in the case of chronic kidney disease and in renal graft recipients, due to having an estimated similar glomerular filtration rate [11]. Anemia may also lead to death in organ recipients, as cardiovascular disease is the main cause of death in renal transplant individuals [12,13]. Moreover, anemia itself is related to an increased death rate in recipients of renal graft [14,15]. It is also important to underline the impact of inflammation prior to and after transplantation on iron absorption and homeostasis, because in inflammatory conditions serum iron may be low, transferrin saturation may be reduced, and the production of hepcidin is induced by the proinflammatory cytokines. Chronic inflammatory conditions are also related to macrophage iron accumulation [5].

The purpose of this article is to analyze the available data on iron metabolism in transplant patients, with a special emphasis on the influence of pharmacotherapy on iron management in transplantology.

## 2. Iron Homeostasis

Due to the lack of physiological excretion mechanisms, the iron content in the body depends on the homeostasis of the metal, which is a result of the absorption of iron in the intestines and the release of iron from red blood cells, which is regulated by specific proteins. It is also important to underline that iron absorption can be affected by gut microbiota or the pH of the colon contents, which may also depend on several factors such as age, ethnicity, geography and even lifestyle. Moreover, inflammatory bowel disease may also impair iron absorption [57]. The key proteins involved in iron metabolism are, among others, transferrin, soluble transferrin receptor (sTFR), ferritin, ferroportin, hepcidin and hemojuvelin (Figure 1) [10].

Hepcidin is a peptide hormone and an acute phase protein. It is produced in the liver and plays a role as a key regulator of iron homeostasis. Its secretion is augmented by inflammation and iron loading [11]. It causes the downregulation of the absorption of iron and the release of recycled hemoglobin iron from macrophages. A high hepcidin level is associated with reduced absorption of iron in the intestine. The binding of hepcidin to ferroportin, a cellular iron export pump, leads to its degradation. The ineffective function of hepcidin may cause the manifestation of human hemochromatosis disorders. Normally, increased iron availability increases the expression of hepcidin. Hepcidin interacts with a transmembrane protein ferroportin and is responsible for the process of absorption, storage and the distribution of the element in human body [58]. The secretion of hepcidin is regulated by iron, which in turn regulates the concentration of ferroportin, thus contributing to iron homeostasis. Moreover, during pregnancy, hepcidin regulates the transfer of the iron across the placenta to the fetus [11].

The overexpression of hepcidin leads to severe iron deficiency anemia, causing death within a few hours after birth in transgenic mice [59]. An increased hepcidin level has been found in kidney and heart transplant recipients [60,61]. The augmented level of hepcidin observed by Przybyłowski et al. [62] in patients with heart failure was associated with compromised erythropoiesis contributing to anemia.

High hepcidin concentrations are also present in dialysis patients [63]. This, in turn, may be consistent with findings that they frequently develop iron deficiency [5,64]. Iron storage in the body and inflammation raise the level of hepcidin, while hypoxia and increased erythropoiesis contribute to a reduced expression of hepcidin, leading to an increased absorption of iron. Hepcidin deficiencies can lead to hemochromatosis, which involves the excessive accumulation of iron in tissues [1,58,59].

Additionally, hemojuvelin plays a significant role in the homeostasis of iron. This protein is a hepcidin regulator which signals to promote the expression of hepcidin. Hemojuvelin bound to membrane inhibits iron absorption in the gut through the promotion of hepcidin synthesis [65]. Mutations in several genes encoding hemojuvelin may also cause iron loading syndromes, resembling hepcidin deficiency [66].

Another peptide, transferrin, delivers iron to cells that require it, mainly to red blood cell progenitors. The bone marrow acquires iron for the production of red blood cells, from macrophages that collect iron from recycled red blood cells. The rest of the iron is stored in the cytosolic iron storage protein, ferritin, in hepatocytes. After the secretion of ferritin into the plasma, it constitutes body iron stores [5,11].

Ferroportin is an iron transporter, which is important during the absorption of iron in intestines and its release from cells. Other cells taking part in iron homeostasis, e.g., duodenal enterocytes, hepatocytes, placental cells and macrophages, also contain ferroportin. The internalization and degradation of ferroportin caused by hepcidin leads to iron trapping within the cells [67].

The inactivation of the murine *ferroportin* gene by Donovan et al. [68] caused the accumulation of iron in hepatocytes, enterocytes and macrophages. Moreover, the inactivation of intestinal ferroportin also explained its contribution to the absorption of iron in the intestine. The global inactivation of ferroportin led to early failure in embryonic development, while the inactivation of ferroportin only selectively in the postnatal intestine caused severe iron deficiency, which could be compensated only by the delivery of iron parenterally. The study by Donovan et al. also confirmed that ferroportin is the main iron exporter that is crucial in iron turnover, not only in epithelial cells, but also in iron-recycling macrophages and hepatocytes.

As shown by Nemeth et al. [59], the cellular iron level depends more on the export of iron from cells by ferroportin than the availability of iron undergoing cell influx. The elevated expression of ferroportin in iron deficiency should secure the augmented absorption of iron in the intestine, respecting the fact that iron absorption from the gut can be also influenced by age, gut condition and microbiome, and can also cause effective regaining of the iron that was transferred to the intestinal epithelium from plasma. The study by Donovan et al. [68] also confirmed that ferroportin is the main iron exporter that is crucial in iron turnover, not only in epithelial cells but also in hepatocytes and in iron-recycling macrophages. The trapping of iron in these cells together with impaired iron absorption deepens the anemia.

sTFR is a protein found on the surface of the cell membrane responsible for the binding of transferrin, a process that regulates the delivery of iron to cells. Then, the liberation of iron from the cells (enterocytes or macrophages) takes place with ferroportin. Since sTFR is not an acute phase protein, its study is an alternative to ferritin in cases of the occurrence or suspicion of chronic disease. When assessing iron homeostasis, it is also important to specify parameters such as TIBC, total iron binding capacity (measured or calculated on the basis of transferrin concentration), and TSAT, which means transferrin saturation calculated on the basis of iron concentration and TIBC [1,2,69]. Summing up, iron uptake requires protein transferrin receptor 1 (TfR1), whereas iron liberation needs protein ferroportin 1 (Fpn1) and iron storage requires ferritin [19].

In iron overload, decreased ferroportin expression leads to an increase in iron loss thanks to the elevation of the iron content, which is accumulated by old enterocytes undergoing excretion to the gut lumen. This seems to be an important mechanism of iron regulation as neither the liver nor kidney had mechanisms of iron removal [68]. Iron overload may be a result of mutations in some genes responsible for iron homeostasis (hereditary hemochromatosis), and may be due to refractory anemias, chronic liver diseases, chronic transfusions [70] and eventually some transplantations [1]. Beta thalassemia and sideroblastic anemia classified as “iron-loading” refractory anemias are connected with ineffective erythropoiesis, erythroid hyperplasia and the excessive absorption of iron [70].

## 3. Redox Activity of Iron

Iron is a transition element which belongs to the d-block and exists in biological systems in oxidation states +2 (ferrous), +1 (ferric) and +4 (ferryl). It binds to several ligands, thanks to its unoccupied d orbitals. Among iron ligands are the atoms of nitrogen, oxygen and sulfur. Iron may change the biological redox potential as well as the electronic spin state, depending on the ligand [71].

The redox status of the graft implicated by iron remains an important factor in the general assessment of the organ prior to the procurement as well as after transplantation. It is known that highly reactive iron may worsen the whole outcome, as it is crucial in the ischemia-reperfusion injury after being released from cells in response to ischemia. Therefore, it has been already well documented that the chelation of iron, e.g., with deferoxamine, is favorable due to the reduction of ischemia-reperfusion injury [5]. Fe^2+^ enters the Fenton reaction and catalyzes the conversion of H_2_O_2_ into cell-toxic radicals, mostly OH· and OH^−^. There is a correlation between the available amount of Fe^2+^ and lipid peroxidation in the liver mitochondrium and microsomes induced by free radicals [72,73]. Moreover, the pulmonary fibrosis is a result of the accumulation of Fe^2+^ on the fiber surface, acting as a Fenton reaction catalyzer [46]. The risk of adverse redox reactions of free iron is increased in individuals suffering from iron overload, and is also connected with an enhanced risk of infection [70].

## 4. Iron and Immune System

The transplantation of a solid organ is a cause of the activation of the immune system, the intensity of which is a result of iron status. The consequent activation of T-cells is attenuated by iron [5]. It has been stated that individuals with iron deficiency anemia may have decreased IgG levels, interleukin 6 (IL-6) and phagocytic activity, and that there is a significant positive correlation between serum level of iron and IL-6 [74].

The process of T-cells’ activation, proliferation and differentiation is mediated through RAS, NF-kappaB and the calcineurin pathway, which are activated by the so called “immunological synapse” formed by CD3, CD71, TfR and the T-cell receptor. The decreased activation of the immune response in iron deficiency is because of attenuated IL-2 levels and CD3 expression. Thus, iron is crucial for the proliferation and differentiation of T-cells. Moreover, a blockade of anti-CD71 antibodies may attenuate T-cell activation due to iron chelation. Similarly, iron deficiency is also connected with reduced CD28 expression, which is also involved in RAS, NF-kappaB and calcineurin pathways [5]. It is also important to underline that the RAS pathway has been found to have the potential to suppress hepcidin expression [5,75].

Iron deficiency diminishes the cellular immune response, whereas iron overload impairs the function of macrophages [5], which remains consistent with the observation of Das et al., according to whom significantly lower levels of CD4+ T-cells as well as reduced CD4:CD8 ratios have been observed among children with iron deficiency. These effects may be improved after iron supplementation [76].

Inflammation suppresses erythropoiesis due to the sequestration of iron from erythroid precursors. It reduces the flow of iron into the circulation, where it can be used by erythroblasts thanks to the binding of TfR1 with transferrin, which leads to anemia in chronic inflammation [67]. The induction of hepcidin by proinflammatory cytokines during inflammation causes a decrease in blood iron level, which is known as hypoferremia of inflammation [59].

Both iron deficiency as well as iron overload are connected with impaired activation of the immune system. Hyperferritemia is also observed in inflammatory conditions, with accompanied lowered transferrin saturation and low serum iron. High intracellular iron in red blood cells may impact their immune-effector properties, and these cells undergo recycling by macrophages. The accumulation of iron in macrophages occurs in chronic inflammation and correlates with an impaired role as an immune effector [5]. Additionally, IL-6 is necessary in the process of hepcidin synthesis in inflammation [1].

Additionally, ferritin, which is a cellular iron storage protein, may be affected by inflammation processes in the body. It is an acute phase protein, which means that its level increases due to inflammation or in the course of various autoimmune and inflammatory diseases, as well as chronic infections [2].

According to Attia et al. [77], there is a difference in the level of appropriate mature or immature T-lymphocytes between individuals with iron deficiency anemia and control subjects. Children with iron deficiency anemia had significantly higher levels of immature T-cells CD1 a(+) as well as lower levels of mature T-lymphocytes CD4(+) and T-lymphocytes CD8(+) when compared to the control, indicating a defect in T-cell maturation in iron deficiency anemia patients.

## 5. Iron and Infections

It is also important to underline the involvement of microorganisms in iron homeostasis in the human body, since biochemical pathways utilized by many pathogens may interfere with iron turnover, causing interactions with drugs and becoming an additional factor that may lead to pharmacotherapy failure in transplant patients.

Iron is a key element for the human body and also for microorganisms, being a good redox catalyst for processes such as DNA replication and respiration. On the other hand, its redox potential is also responsible for its toxicity. The pathogens may cause disease after overcoming the so-called nutritional immunity, which is a process of limiting the access to iron by the immune system [70]. Hypoferremia is supposed to increase the resistance of the host organism to microbial infection; on the other hand, it may lead to anemia of inflammation [59].

Iron is an important nutrient for many human bacterial pathogens, which have developed different mechanisms of iron’s acquisition such as the production of iron transporters, siderophores (small ferric iron chelators capable of binding iron), heme acquisition systems, and receptors for transferrin and lactoferrin. Siderophores may compete with host transferrin that also binds iron, by binding iron with very high association constants [70]. Siderophores have the ability to take up other iron-chelating substances, so it is important to take into consideration that the treatment of iron overload with such molecules as desferrioxamine may be connected with the increased risk of infection [78]. Siderophores are produced by *Escherichia coli*, *Staphylococcus aureus*, *Bacillus anthracis* and *Legionella pneumophila* [79].

Other mechanisms responsible for iron acquisition by pathogenic microbes are heme uptake systems from erythrocytes. This strategy is used, among others, by *Haemophilus influenzae*, which is incapable of endogenous heme biosynthesis, *Pseudomonas aeruginosa*, *Pseudomonas fluorescens*, *S. aureus*, *Yersinia pestis* and *Yersinia enterocolitica*. Moreover, pathogens also developed iron acquisition mechanisms involving transferrin/lactoferrin receptors. These are *Neisseria meningitides* [70,80].

Predominantly, intracellular bacteria try to acquire iron from macrophages. *Mycobacterium tuberculosis*, after being phagocytosed by alveolar macrophages, obtains host iron. Additionally, pathogenic fungi have developed different mechanisms for the acquisition of iron from host tissues, such as uptake mediated by siderophores, reductive uptake, and heme acquisition [70]. *Saccharomyces cerevisiae*, *Cryptococcus neoformans* and *Candida albicans* contain ferric reductases. In the process of ferric reduction to ferrous iron, the element shifts from the host molecules that chelate it into the fungal cell. Many pathogenic fungi produce siderophores (*Blastomyces dermatitidis*, *Histoplasma capsulatum*, *Aspergillus* spp. and additionally *Rhizopus* spp.) [81]. The heme uptake is also needed for the growing of *C. albicans*, *C. neoformans*, and *H. capsulatum* [70].

Many viruses, such as human immunodeficiency virus (HIV), hepatitis B virus (HBV), hepatitis C virus (HCV), parvovirus B19 and Epstein–Barr virus, can interfere with myelopoiesis and cause aplastic anemia [82,83]. Parvovirus B19 causes anemia by replicating in erythroid precursors, which proliferate and lead to red blood cell aplasia due to the induction of apoptosis in their precursors [84]. Similarly, Epstein–Barr virus is thought to cause anemia by infecting bone marrow progenitor cells [71].

## 6. Pharmacotherapy and Iron Turnover in Transplant Patients

The course of iron metabolism can be influenced by the pharmacotherapy. Taking into consideration that pre- and post-transplant patients have to pharmacologically maintain appropriate immune responses, antimicrobial resistance and good graft parameters should avoid the worsening of many other coexisting illnesses; they use various drugs from many different pharmacological groups that often interfere with mechanisms responsible for iron turnover, and may lead to the occurrence of anemia or iron overload.

The use of pharmacotherapy may influence, among other things, the action of hepcidin, transferrin and ferritin and may lead to the chelation of iron or a change in iron’s disposition in the human body. The complex evidence for the link between pharmacotherapy used by transplant patients, iron metabolism and anemia has not been studied so far, probably due to a lack of systematic data. Our analysis is presented in Table 1.

It may be possible that certain drugs will directly interact with iron. So-called pharmaceutical interactions between drugs and iron may lead to the formation of complexes that limit the availability of free iron. This is in the case for acetylsalicylic acid [16,17]. Antiviral drugs also have influence on iron turnover, by binding to endogenous or exogenous iron [26].

Drugs may also impact the level of key proteins involved in iron turnover. Reports from studies on cell cultures reveal that acetylsalicylic acid also has the potential to decrease the level of hepcidin and induce the production of ferritin [18,20]. Similarly, simvastatin as well as vitamin C may suppress the expression of hepcidin in cell cultures [21,22].

Cellular iron levels can be affected by immunosuppressive drugs. Data on animal models show that sirolimus may contribute to iron loss on a cellular level [24], which also seems important in cases of human models where the use of sirolimus was correlated with functional iron deficiency and anemia [28,29,85,86]. Similarly, therapy with everolimus and tacrolimus has been found to be associated with the occurrence of anemia [32]. Additionally, cyclosporine may contribute to anemia, as has been stated regarding the liver and lung in transplant patients [34]. Sirolimus, by its mechanism of immunosuppression, blocks the IL-2 post-receptor signals mediating T-cell proliferation, having the ability to bind to the mammalian target of rapamycin and arrest the progression of the cell cycle between the G1 and the S phase [86]. The study of Thaunat et al. indicated that anemia in sirolimus therapy is associated with low serum iron levels, inflammatory states and high serum ferritin levels [31]. Some authors demonstrated that sirolimus-induced anemia occurs due to its direct impact on the homeostasis of iron [30]. On the other hand, introducing prednisone may attenuate the severity of anemia in individuals after renal transplantation. Additionally, treatment with mycophenalate mofetil was associated with the occurrence of anemia in both rena and liver transplant patients [37].

Iron uptake is also a process that can often be influenced by pharmacotherapy. The significant finding by Kramer et al. [27] in an animal model could also be of interest in the case of heart transplantology in patients taking propranolol, as it may reduce iron uptake by cardiac tissue in rats. Regarding statins, pravastatin increases iron uptake in the liver [25] while fluvastatin treatment may decrease serum levels of hepcidin prohormone (prohepcidin) [53]. On the other hand, simvastatin has a minor influence on serum prohepcidin levels, hs-CRP or IL-6, which also take part in iron turnover [54].

The non-specific way in which drugs impact iron turnover can be observed in cardiac and dialysis patients. In renal transplant patients, the use of enalapril was associated with anemia [38]. Another hypertensive drug, amlodipine, was reported to decrease iron overload and to reduce ferritin levels in patients with thalassemia major [47]. Nonsteroidal anti-inflammatory drugs (NSAIDs) (loxoprofen, diclofenac, ampiroxicam, naproxen, etodolac) are supposed to increase the risk of iron deficiency in chronic hemodialysis patients, according to Wang et al. [52]. However, acetylsalicylic acid in a representative cohort did not affect serum iron or iron saturation [48]. Nevertheless, treatment with acetylsalicylic acid was related to lower serum levels of ferritin in a group of elderly subjects [50], in postmenopausal women [51], as well as in *H. pylori* infected subjects [87]. Azathioprine was reported by Habas et al. [44] to cause an increase of serum iron and serum transferring saturation in post-kidney transplantation.

Drug-mediated protein expression may be also involved in iron homeostasis. According to Mleczko-Sanecka et al. [75], the suppression of hepcidin is strongly related to mTOR signaling, since the inhibition of the mTOR kinase with rapamycin may lead to a strong activation of the expression of hepcidin mRNA. Successful treatment of the anemic state by intravenous iron, with simultaneous failure of oral therapy, may suggest that the reason is a hepcidin-mediated reduction of iron absorption from diet [75]. Rapamycin and cycloheximide act by blocking protein synthesis, but cycloheximide may impair the process of translation faster in comparison to rapamycin [49]. Interestingly, in the presence of cycloheximide acting as a translation elongation inhibitor, ferroportin remained on the cell surface while only hepcidin impaired the functioning of ferroportin by causing its internalization [59].

Drugs may also take part in the regulation of iron homeostasis, by modifying the effectiveness of the iron transfer according to the needs of the cell, in the case of restricted iron availability, which is supposed to occur in the mTOR pathway. It is supposed that the mTOR target, tristetraprolin (TTP), may alter the level of TfR1 [88]. Mice treated with rapamycin mediated mTOR inhibitors exhibited augmented levels of TTP and reduced levels of TfR1. This resulted in increased levels of iron and ferritin, with a reduction of cellular iron uptake [24]. It has been also confirmed by Przybyłowski et al. [89] that the treatment of heart transplant recipients with mTOR inhibitors was related to increased concentrations of circulating hepcidin, which may be a reason for the observed lower hemoglobin levels.

## 7. Pharmacotherapy and Different Types of Anemia in Transplant Patients

**Figure 2:**
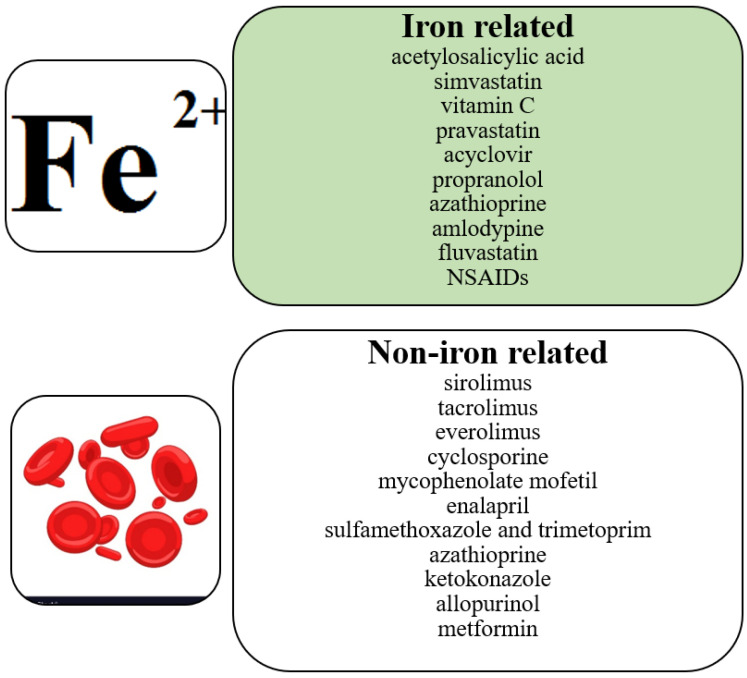
Correlation between pharmacotherapy and anemia.

Post-transplant anemia is multifactorial. Iron deficiency anemia, on which we focus, is the most common cause of anemia in the world. Some drugs cause anemia in the iron deficiency pathway (Table 1). Other drugs affect red blood cells by causing different types of anemia: megaloblastic anemia, aplastic anemia, hemolytic anemia, thrombocytopenia and agranulocytosis [90] (Figure 2). Finally, it has to be taken into consideration that the activity of several drugs may be also driven by inflammatory conditions that may additionally complicate pharmacotherapy prior to and after organ transplantation.

### 7.1. Megaloblastic Anemia

**Figure 3:**
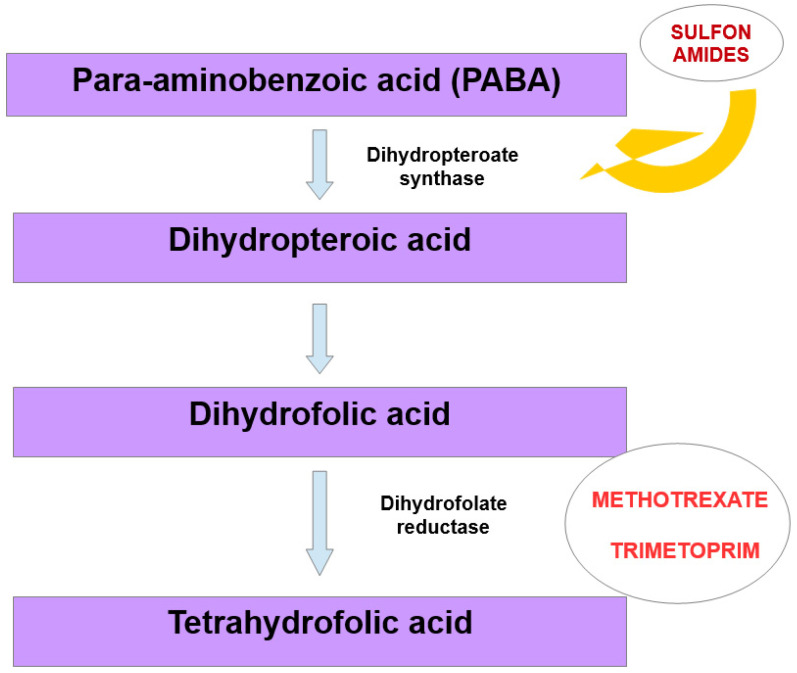
Mechanism that may lead to megaloblastic anemia, due to structural similarity between bacterial and human dihydrofolate reductase.

Megaloblastic anemia is a disease in which an unproductive hematopoiesis occurs, usually from a deficiency of folic acid and/or vitamin B12 as well as from a metabolic deficiency [91]. This type of anemia can be caused by drugs via decreasing the absorption of folic acid, having folate analogue activity, interfering with pyrimidine synthesis, modulating purine metabolism, decreasing the absorption of vitamin B12 or destroying vitamin B12 [92,93]. Among these drugs are sulfonamides. They are structurally related to *para*-aminobenzoic acid, and are its competitive antagonists. Bacteria need *para*-aminobenzoic acid for the formation of dihydrofolic acid, which is required for the synthesis of folic acid. Folic acid is a substrate for nucleic acid synthesis. Sulfonamides are not toxic to human cells, as they utilize folic acid from the diet, but the long-term use of sulfonamides may cause metabolic deficiency that can result in megaloblastic anemia [94]. Trimetoprim, on the other hand, inhibits the reduction of dihydrofolic acid to tetrahydrofolic acid by binding to dihydrofolate reductase. Tetrahydrofolic acid is then needed for the synthesis of thymidine, and the inhibition of this pathway impairs bacterial DNA synthesis (Figure 3). In this way, co-trimoxazole therapy (sulfamethoxazole and trimethoprim) inhibits two related reactions which are crucial for microorganisms [95].

Methotrexate is used for the treatment of leukemias and lymphomas, for auto-immune diseases such as rheumatoid arthritis, and in transplant patients for immunosuppression and for inhibition of inflammation [96,97]. In the context of anemia, it is important to underline that human and bacterial dihydrofolate reductases are structurally similar, and their impairment in mammalian cells may cause megaloblastic anemia (Figure 3).

Phenytoin belongs to anticonvulsant drugs and is used for the treatment of various types of seizures. It may increase the pH in the small intestine and impair the intestinal conjugate activity, leading to decreased intestinal absorption of folates. It directly competes with folates for their uptake sites. Moreover, phenytoin may reduce the activity of folate inter-converting enzymes and increase the activity of enzymes that catabolize folates. Because of this, and additionally by inhibiting central appetite centers, the drug may cause folate deficiency [98].

Azathioprine, an immunosuppressive drug used for the treatment of rheumatoid arthritis, Crohn’s disease, ulcerative colitis and, in transplantology, as a prevention of kidney transplant rejection, inhibits converting reactions among the precursors of purines and suppresses the synthesis of purines [99]. This may result in a deficiency of erythroid precursors from the bone marrow [100]. Similarly, mycophenolate mofetil is another purine synthesis inhibitor. This drug, used in transplantology to prevent graft rejection, impairs the synthesis of purines by blocking the inositol monophosphate dehydrogenase [101]. Nitrous oxide, an inhalatory gas used in anesthesia, may impair the conversion of the reduced form of vitamin B12 to the oxidized form, and thus may also lead to megaloblastic anemia. Since methionine synthase utilizes vitamin B12 in a reduced form (methylcobalamin) in order to transform homocysteine to methionine, and in mitochondria vitamin B12 in the oxidized form (5′-deoxyadenosylcobalamin) is needed for the conversion of methylmalonylcoenzyme A (CoA) to succinyl CoA, nitrous oxide may lead to the impairment of methylation reactions and DNA synthesis [102]. On the other hand, drugs that have an impact on vitamin B12 absorption are neomycin, metformin, aminosalicylic acid and colchicine, but the risk of inducing megaloblastic anemia by them is rather low [56].

### 7.2. Aplastic Anemia

There are two classes of aplastic anemia: inherited and acquired aplastic anemia. Acquired aplastic anemia may be induced by chemical exposure, viral infections, exposure to radiation and drugs [103]. Aplastic anemia induced by drugs involves the generation of intermediate metabolites, binding to proteins and DNA, which causes toxic effects on bone marrow and hematopoietic cells (carbamazepine, captopril, furosemide, thiazides, sulfonamides, chlorothiazide and lisinopril) [104]. Additionally, immune-mediated mechanisms as well as direct toxicity may be involved in drug-induced acquired aplastic anemia [105]. Angiotensin converting enzyme inhibitors (ACEIs) such as lisinopril or captopril may have an impact on the production of erythropoietin (EPO) and cause its inhibition, while angiotensin II is known to augment the proliferation of erythroid progenitors [105]. Moreover, carbamazepine, an anticonvulsant used for the treatment of epilepsy and pain associated with neuralgia, exhibits many side effects which include hematopoietic disorders, such as aplastic anemia. Since the mechanism of these side effects has not been explained so far, the involvement of some toxic effects or allergic reactions has been postulated [106,107].

### 7.3. Hemolytic Anemia

Hemolytic anemia in the drug-induced form may be mediated by metabolic abnormalities in red blood cells, as well as by the production of antibodies. The specific form of anemia called oxidative hemolytic anemia may be caused by glucose-6-phosphate dehydrogenase deficiency, reduced activity of methemoglobin reductase and glutathione peroxidase. Drugs that may induce oxidative hemolytic anemia are nitrofurantoin, sulfacetamide, metformin or ascorbic acid [93], tacrolimus, ciprofloxacin, ceftriaxone and omeprazole. The mechanism may be due to drugs attaching to the surface of red blood cells, resulting in hemolysis. Moreover, drugs may induce changes in the red blood cells’ membrane. Another one is the mechanism in which antibodies are developed against drug complexes. The complexes bind to the surface of red blood cells, and impair their functionality [108]. Cephalosporins belonging to β-lactam antibiotics are able to interact with the red blood cells’ membrane. First generation cephalosporins may induce hemolysis by causing IgG adsorption to the red blood cells’ membrane, which in turn may result in hemolysis. Ceftriaxone is supposed to induce an immune-complex reaction, where IgM antibodies against ceftriaxone are involved, leading to erythrocyte destruction [42,109]. Additionally, chloroquine, an antimalarial drug and a second line treatment for rheumatoid arthritis, as well as nitrofurantoin, an antibiotic used mainly for the treatment of urinary tract infections, may cause the development of hemolytic anemia when the functionality of glucose-6-phosphate dehydrogenase (G6 PD) is impaired, thus becoming unable to protect red blood cells from certain oxidative metabolites and stressors [110,111].

## 8. Conclusions and Perspectives

Summarizing, anemia caused by drugs directly affecting red blood cells is described widely in the literature, while the problem of iron metabolism and transplantation has been mentioned in the literature by several authors to date. Although the metabolic pathways of iron in the human body are well described, the influence of the transplantation of organs, as well as perioperative pharmacotherapy, which both seem to affect the homeostasis of iron in a very diverse way, are not studied enough.

After organ transplantation, iron deficiency occurs often in patients with renal and cardiac grafts. However, in patients after hepatic transplantation, iron overload is more common [1]. In most cases, authors reviewed the metabolism of iron in kidney, liver and heart transplantations, but the current knowledge about iron metabolism in lung graft recipients and donors is limited. Nevertheless, the overall significance of iron turnover among patients before and after transplantation seems to have hardly been discussed so far and surely needs more attention.

Since iron turnover may be impaired by so many factors, more clinical data on drugs safety related to iron status prior to and after transplantation is needed. This knowledge would be helpful in predicting the risk of iron deficiency and overload related to pharmacotherapy in transplant patients. Moreover, the course of management of patients with iron overload seems to remain a challenge, as there are no evidence-based recommendations for post-transplant patients.

## Figures and Tables

**Table 1:** The link between pharmacotherapy commonly used in transplantology and anemia.

Drug	Model	Link with Anemia	Data Source
In vitro			
**Acetylsalicylic acid**	Pharmaceutical interaction	Formation of drug–iron complexes (further study needed to assess the biological significance)	Zhang et al. [16]
	Pharmaceutical interaction	Acetylsalicylic acid may chelate endogenous hepatic iron	Schwarz et al. [17]
*Cell cultures*			
**Acetylsalicylic acid**	Microglial cells under inflammatory conditions	Downregulation of hepcidin by inhibition of NF-κB and IL6/JAK2/STAT3 pathways	Li et al. [18]
	Microglial cells under inflammatory conditions	Negative effect on cell iron contents under ‘normal’ conditions, potential to partly reverse iron imbalance under inflammatory conditions	Xu et al. [19]
	Bovine pulmonary artery endothelial cells	Aspirin at low antithrombotic concentrations induced the synthesis of ferritin protein in a time- and concentration-dependent fashion	Oberle et al. [20]
**Simvastatin**	HepG2 cell line (human liver carcinoma cell line)	Simvastatin significantly suppressed the mRNA expression of hepcidin	Chang et al. [21]
**Vitamin C**	HepG2 cell line (human liver carcinoma cell line)	Vitamin C directly inhibits hepcidin expression	Chiu et al. [22]
*Animal model*			
**Sirolimus**	Healthy rats	Microcytosis and polyglobulia	Diekmann et al. [23]
	Mice	Export-dependent iron-loss on cellular level	Bayeva et al. [24]
**Pravastatin**	Rat model of cholestasis	Pravastatin raised liver iron content by modulation of heme catabolism and an increase in hepatic iron uptake and storage capacity.	Kolouchova et al. [25]
**Acyclovir**	Rats	Acyclovir binds endogenous and exogenous iron	Müller [26]
**Propranolol**	Rats	Reduction of cardiac tissue iron uptake	Kramer et al. [27]
*Human model*			
**Sirolimus**	Heart transplant patients	Anemia of chronic disease and functional iron deficiency	McDonald et al. [28]
	Renal transplant patients	Anemia, red blood cell microcytosis	Sofroniadou et al. [29]
	Renal transplant patients	Anemia	Maiorano et al. [30]
	Renal transplant patients	Anemia due to defective IL-10 dependent inflammatory regulation	Thaunat et al. [31]
**Everolimus**	Renal transplant patients	Anemia, microcytosis	Sánchez Fructuoso et al. [32]
	Liver transplant patients	Anemia	Masetti et al. [33]
**Cyclosporine**	Liver transplant patients	Anemia	Masetti et al. [33]
	Liver transplant patients	Hemolytic anemia (microangiopathic hemolytic anemia—MAHA) related to injury of microvascular endothelial cells and apoptosis	Kanellopoulou et al. [34]
**Cyclosporine/** **Sirolimus/** **Steroids**	Renal transplant patients	Cyclosporine withdrawal followed by sirolimus immunotherapy resulted in significantly less anemia than sirolimus-cyclosporine-steroids therapy	Friend et al. [35]
**Tacrolimus**	Liver and renal transplant patients	Hemolytic anemia (microangiopathic hemolytic anemia—MAHA) related to injury of microvascular endothelial cells and apoptosis	Kanellopoulou et al. [36]
**Prednisone**	Renal transplant patients	Pro-erythropoietic, steroid therapy may reduce the severity of anemia in the early post-transplant period	Al-Uzri et al. [37]
**Mycophenolate mofetil**	Renal transplant patients	Megaloblastic anemia	Al-Uzri et al. [37]
	Liver transplant patients	Anemia	Al-Uzri et al. [37]
**Enalapril**	Renal transplant patients	Anemia	Vlahakos et al. [38]
	Renal transplant patients	Anemia	Graafland et al. [39]
**Sulfamethoxazole and trimethoprim**	Single patient	Drug-induced immune hemolytic anemia	Frieder et al. [40] Arndt et al. [41]
	Single patient	Drug-induced immune hemolytic anemia with antibodies to both substances	Arndt et al. [42]
	Single patient	Hemolytic anemia	Chisholm-Burns et al. [43]
**Ketoconazole, fluconazole**	-	-	No data
**Azathioprine**	Post-kidney transplantation	Serum iron and serum transferrin saturation increased significantly. No evidence of hemolysis	Habas et al. [44]
**Azathioprine**	Post-liver transplantation	Modulation of purine metabolism	Maheshwari et al. [45]
**Azathioprine and prednisolone**	Single patient	Successful management of idiopathic pulmonary hemosiderosis	Willms et al. [46]
**Basiliximab**	-	-	No data
**Thymoglobulin**	-	-	No data
**Rituximab**			
**Amlodipine**	Patients with thalassemia major	Amlodipine decreases iron overload and reduces ferritin levels	Fernandes et al. [47]
**Acetylsalicylic acid**	A representative cohort of community-dwelling subjects	No association between aspirin use and reduced serum iron or iron saturation	Hammerman-Rozenberg et al. [48]
	Males and females infected or not infected by *H. pylori*	No effect of low-dose aspirin use on ferritin levels (in males); lower ferritin levels in *H. pylori* infected subjects using aspirin, compared with both uninfected and infected non-aspirin users (in females)	Kaffes et al. [49]
	Elderly participants	Aspirin use is associated with lower serum ferritin	Fleming et al. [50]
	Postmenopausal women	19% lower mean serum ferritin in aspirin users than in non-users	Liu et al. [51]
**NSAIDs (loxoprofen, diclofenac, ampiroxicam, naproxen, etodolac)**	Patients undergoing chronic hemodialysis	The use of non-aspirin NSAIDs may increase the risk of iron deficiency	Wang et al. [52]
**Fluvastatin**	Dyslipidemic end-stage renal disease patients with renal anemia	Fluvastatin treatment decreased high-sensitive C-reactive protein (hs-CRP) and serum prohepcidin (prohormone of hepcidin) levels	Arabul et al. [53].
**Simvastatin**	End-stage renal disease patients with renal anemia	Simvastatin did not significantly change the serum prohepcidin, hs- CRP, or IL-6 concentrations	Li et al. [54]
**Valgancyclovir**	-	-	No data
**Allopurinol**	Patient with chronic kidney disease	Aplastic anemia	Kim et al. [55]
**Metformin**	Patients treated with metformin	Reduction of vitamin B12	Liu et al. [56]

## Data Availability

Not applicable.
